# *Drosophila* Yemanuclein and HIRA Cooperate for *De Novo* Assembly of H3.3-Containing Nucleosomes in the Male Pronucleus

**DOI:** 10.1371/journal.pgen.1003285

**Published:** 2013-02-07

**Authors:** Guillermo A. Orsi, Ahmed Algazeery, Régis E. Meyer, Michèle Capri, Laure M. Sapey-Triomphe, Béatrice Horard, Henri Gruffat, Pierre Couble, Ounissa Aït-Ahmed, Benjamin Loppin

**Affiliations:** 1 Centre de Génétique et de Physiologie Moléculaire et Cellulaire, CNRS UMR5534, Université Claude Bernard Lyon 1, Villeurbanne, France; 2 Institut de Génétique Humaine, CNRS UPR 1142, Montpellier, France; 3 Laboratoire de Biologie Moléculaire des Herpesvirus, INSERM U758, Ecole Normale Supérieure de Lyon, France; University of Cambridge, United Kingdom

## Abstract

The differentiation of post-meiotic spermatids in animals is characterized by a unique reorganization of their nuclear architecture and chromatin composition. In many species, the formation of sperm nuclei involves the massive replacement of nucleosomes with protamines, followed by a phase of extreme nuclear compaction. At fertilization, the reconstitution of a nucleosome-based paternal chromatin after the removal of protamines requires the deposition of maternally provided histones before the first round of DNA replication. This process exclusively uses the histone H3 variant H3.3 and constitutes a unique case of genome-wide replication-independent (RI) *de novo* chromatin assembly. We had previously shown that the histone H3.3 chaperone HIRA plays a central role for paternal chromatin assembly in *Drosophila*. Although several conserved HIRA-interacting proteins have been identified from yeast to human, their conservation in *Drosophila*, as well as their actual implication in this highly peculiar RI nucleosome assembly process, is an open question. Here, we show that Yemanuclein (YEM), the *Drosophila* member of the Hpc2/Ubinuclein family, is essential for histone deposition in the male pronucleus. *yem* loss of function alleles affect male pronucleus formation in a way remarkably similar to *Hira* mutants and abolish RI paternal chromatin assembly. In addition, we demonstrate that HIRA and YEM proteins interact and are mutually dependent for their targeting to the decondensing male pronucleus. Finally, we show that the alternative ATRX/XNP-dependent H3.3 deposition pathway is not involved in paternal chromatin assembly, thus underlining the specific implication of the HIRA/YEM complex for this essential step of zygote formation.

## Author Summary

Chromosome organization relies on a basic functional unit called the nucleosome, in which DNA is wrapped around a core of histone proteins. However, during male gamete formation, the majority of histones are replaced by sperm-specific proteins that are adapted to sexual reproduction but incompatible with the formation of the first zygotic nucleus. These proteins must therefore be replaced by histones upon fertilization, in a replication-independent chromatin assembly process that requires the histone deposition factor HIRA. In this study, we identified the protein Yemanuclein (YEM) as a new partner of HIRA at fertilization. We show that, in eggs laid by *yem* mutant females, the male pronucleus fails to assemble its nucleosomes, resulting in the loss of paternal chromosomes at the first zygotic division. In addition, we found that YEM and HIRA are mutually dependent to perform chromatin assembly at fertilization, demonstrating that they tightly cooperate *in vivo*. Finally, we demonstrate that the replication-independent chromatin assembly factor ATRX/XNP is not involved in the assembly of paternal nucleosomes. In conclusion, our results shed new light into critical mechanisms controlling paternal chromosome formation at fertilization.

## Introduction

Assembly of octameric nucleosomes in eukaryotic chromatin is a stepwise process where deposition of a histone H3-H4 heterotetramer precedes incorporation of two H2A-H2B dimers [1]. While the bulk of *de novo* chromatin assembly occurs during genome replication and mainly involves canonical histone H3, alternative, replication-independent (RI) chromatin assembly pathways use the conserved histone H3 variant H3.3 [2], [3]. Canonical (or replicative) H3s (H3.1 and H3.2 in mammals, H3.2 in *Drosophila*) are synthesized in early S phase and deposited at DNA replication forks by the trimeric CAF-1 (Chromatin Assembly Factor-1) complex [4]. In contrast, H3.3 is expressed throughout the cell cycle and is deposited at various genomic regions in a DNA-synthesis independent manner [5]–[8]. During the past decade, research on H3.3 has largely focused on the ability of this histone to be deposited at transcribed genes, opening the possibility that H3.3 could constitute an epigenetic mark of active chromatin [9]–[13]. Recent advances in the field have let emerge a more complex view of H3.3 biology. Although H3.3 is indeed enriched at transcribed gene bodies, it is now established that this histone is also deposited at various chromatin regions, such as regulatory elements, mammalian telomere repeats or satellite DNA blocks [5]–[7], [14]–[18]. This surprising versatility of H3.3 could simply reflect its ability to be deposited in regions that are subjected to nucleosome depletion or rapid histone turnover [5], [7], [19].

In metazoa, H3.3 is also implicated in a variety of nuclear processes that specifically occur in germ cells and in early embryos [7], [20]–[22]. In mouse spermatocytes, for instance, H3.3-containing nucleosomes are assembled on sex chromosomes during their inactivation and accumulate over the whole sex body [23]. Moreover, an insertion mutation in the mouse *H3.3A* gene induces male subfertility, among other phenotypes [24]. Certain lysine residues of H3.3 are also important for the establishment of heterochromatin during reprogramming in mouse zygotes [25]. Recently, knock-down experiments in *Xenopus laevis* demonstrated a specific and critical requirement of H3.3 during embryo gastrulation [26]. In *Drosophila*, H3.3 deficient animals are viable but are both male and female sterile [27], [28]. H3.3 is notably required for the proper segregation of meiotic chromosomes in spermatocytes [28] and for the global organization of early spermatid chromatin [28], [29].

A remarkable H3.3 deposition process also occurs during the decondensation of the male pronucleus at fertilization [21]. This unique, genome-wide assembly of H3.3 nucleosomes follows the rapid removal of sperm-specific nuclear basic proteins (SNBPs) from the fertilizing sperm nucleus, after its delivery in the egg cytoplasm. In many animal species, during spermiogenesis, histones are progressively replaced with SNBPs, such as the well-characterized protamines [30]–[32]. The nature and extent of this replacement is highly variable in metazoans [32]. In *Drosophila*, protamine-like proteins are encoded by two paralogous genes named *Mst35Ba* and *Mst35Bb* [33], [34]. In this species, the vast majority of sperm DNA is packaged with protamines and with other non-histone SNBPs [21], [35], implying that *de novo* assembly of paternal nucleosomes at fertilization after SNBP removal must occur over the entire male genome.

We had previously shown that this unique RI assembly requires the conserved H3.3 histone chaperone HIRA [36], [37]. Indeed, loss of function mutations in *Hira* are viable in *Drosophila*, but nucleosome assembly in the male pronucleus is completely abolished in eggs laid by mutant females, resulting in the loss of the paternal set of chromosomes and the development of gynogenetic haploid embryos [36], [37]. In mice, HIRA is present in the decondensing male nucleus [38] and is most likely responsible for the strong paternal H3.3 enrichment observed in the zygote [38], [39]. Recently, HIRA has been implicated in the formation of the male pronucleus in the crucian carp [40], confirming the widespread role of this histone chaperone in paternal nucleosome assembly at fertilization.

The Hir/HIRA complex is composed of a small number of proteins that are conserved between yeast and human. In *S. cerevisiae*, the Hir chromatin assembly complex includes the HIRA-related proteins Hir1 and Hir2, Asf1 (Anti Silencing Factor 1), Hir3 and Hpc2 [41]–[43]. Hir3 is a poorly conserved protein related to Hip3 (*S. pombe*) and human CABIN1, but which does not seem to have an ortholog in *Drosophila* [43]–[45]. Hpc2 is functionally related to Hip4 in fission yeast and to the HIRA-associated proteins Ubinuclein 1 and Ubinuclein 2 (UBN1/UBN2) [8], [44], [46]–[48]. Interestingly, the strongest conservation between Hpc2 orthologs resides in a ∼50 amino-acid domain called HRD (Hpc2-Related Domain) or HUN (Hpc2-Ubinuclein-1) domain [44], [48] and to a smaller domain called NHRD [49]. In *Drosophila*, Yemanuclein (YEM; also named Yemanuclein-α [50], [51]) is the only protein with a HRD domain [44]. The *yem* gene has a strong ovarian expression and encodes a nuclear protein that accumulates in the germinal vesicle of growing oocytes [51]. Recently, a mutant allele of *yem* (*yem^1^*) has been characterized as a V478E replacement, which results in female sterility [52]. In this first report on YEM function, YEM was implicated in the segregation of chromosomes during the first female meiotic division but the sterility of mutant females suggested the existence of yet unknown roles for YEM [52]. In this paper, we have explored the implication of YEM in HIRA-dependent RI nucleosome assembly in the zygote. We show that the cooperation of YEM and HIRA *in vivo* is critical for the assembly of H3.3-containing nucleosomes in the male nucleus at fertilization.

## Results

### *yem^2^* is a deletion allele of the *yemanuclein* gene

**Figure 1:**
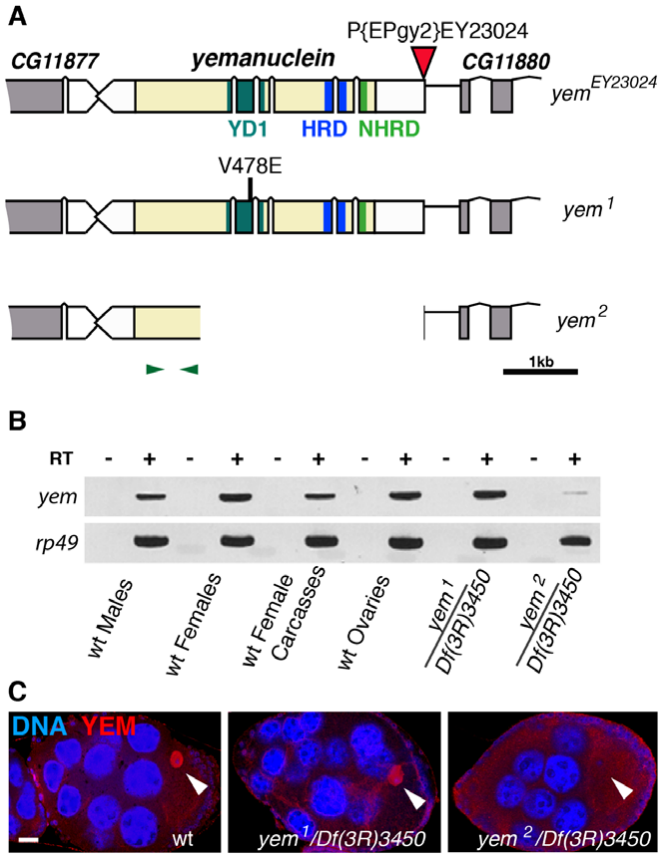
Mutations affecting the *yem* gene. (A) Schematic representation of the *yem* gene [51] and mutant alleles. *yem^1^* is a point mutation (V478E) [52] and *yem^2^* is a deletion that was generated by mobilizing the P-element insertion *P{EPgy2}EY23024* (red triangle). Coding sequence is in yellow and untranslated regions are in white. The YD1 [52], HRD/HUN [44], [48] and NHRD [49] domains of YEM are indicated, as well as the position of primers used for RT-PCR analysis (green arrowheads). (B) RT-PCR analysis of *yem* expression in the indicated tissues and genotypes. RT-PCR amplification used the primer pair shown in (A). (C) Confocal images of wild-type or *yem* mutant egg chambers stained for DNA (blue) and with anti-YEM AS2 antibody (red). YEM protein accumulates in the germinal vesicle (arrowhead) in wild-type and *yem^1^/Df(3R)3450* oocytes. In *yem^2^/Df(3R)3450* mutant oocytes, only background staining is detected. Bar: 20 µm.

The original *yem^1^* point mutation causes a single amino-acid replacement (V478E) in YEM protein (Figure 1A) [52]. This mutation induces female sterility but has no detectable effect on the level of *yem* transcripts in ovaries nor on the accumulation of YEM protein in the oocyte nucleus (or germinal vesicle, GV) (Figure 1B, 1C). To obtain a more severe mutant allele of *yem*, we mobilized a P-element inserted near the transcriptional start site of the *yem* gene (Figure 1A). One of the imperfect excisions of this P-element generated a 3180 bp deletion (named *yem^2^*) that spans the 5′ UTR and most of the coding region of *yem*. Accordingly, the *yem^2^* allele induced female sterility in association with *yem^1^* or with the large non-complementing deficiency *Df(3R)3450* (Table 1). In *yem^2^*/*Df(3R)3450* females, *yem* transcripts (corresponding to a region of the gene not covered by the *yem^2^* deletion) were greatly reduced compared to *yem^1^*/*Df(3R)3450* females, and the YEM protein was not detected in the oocyte nucleus (Figure 1B, 1C). Finally, the female sterility of both *yem* mutant alleles was rescued by expressing a transgenic YEM protein tagged in its C-terminus with the Flag peptide (YEM-Flag) (Table 1). Taken together, these data suggest that *yem^2^* is a null or at least a strong loss of function allele of *yem*.

**Table 1:** Female sterility associated with *yem* mutations.

Genotype of females	No. of eggs	No. of larvae	Hatch rate (%)
*w; yem^EY23024^/Df(3R)3450*	885	758	85.6
*w; yem^1^/Df(3R)3450*	984	0	0
*w; yem^2^/Df(3R)3450*	948	0	0
*w; yem^1^/yem^2^*	1109	0	0
*w yem-flag^HPF1^/w; yem^1^/Df(3R)3450*	1444	8	0.5
*w yem-flag^HPF1^/w; yem^2^/Df(3R)3450*	1337	0	0
*w yem-flag^HPF1^/w yem-flag^HPF1^; yem^1^/Df(3R)3450*	513	362	70.5
*w yem-flag^HPF1^/w Yem-flag^HPF1^; yem^2^/Df(3R)3450*	577	256	44.4
*w; yem-flag^HPF16^/+; yem^2^/Df(3R)3450*	638	507	79.5
*w; yem-flag^HPF16^/yem-flag^HPF16^; yem^2^/Df(3R)3450*	667	660	98.9

All *yem* mutant alleles are described in Figure 1. *Df(3R)3450* is a large deficiency covering the *yem* locus. *yem-flag^HPF1^* and *yem-flag^HPF16^* are two independent insertions of the same transgene.

### YEM interacts with HIRA *in vivo*

**Figure 2:**
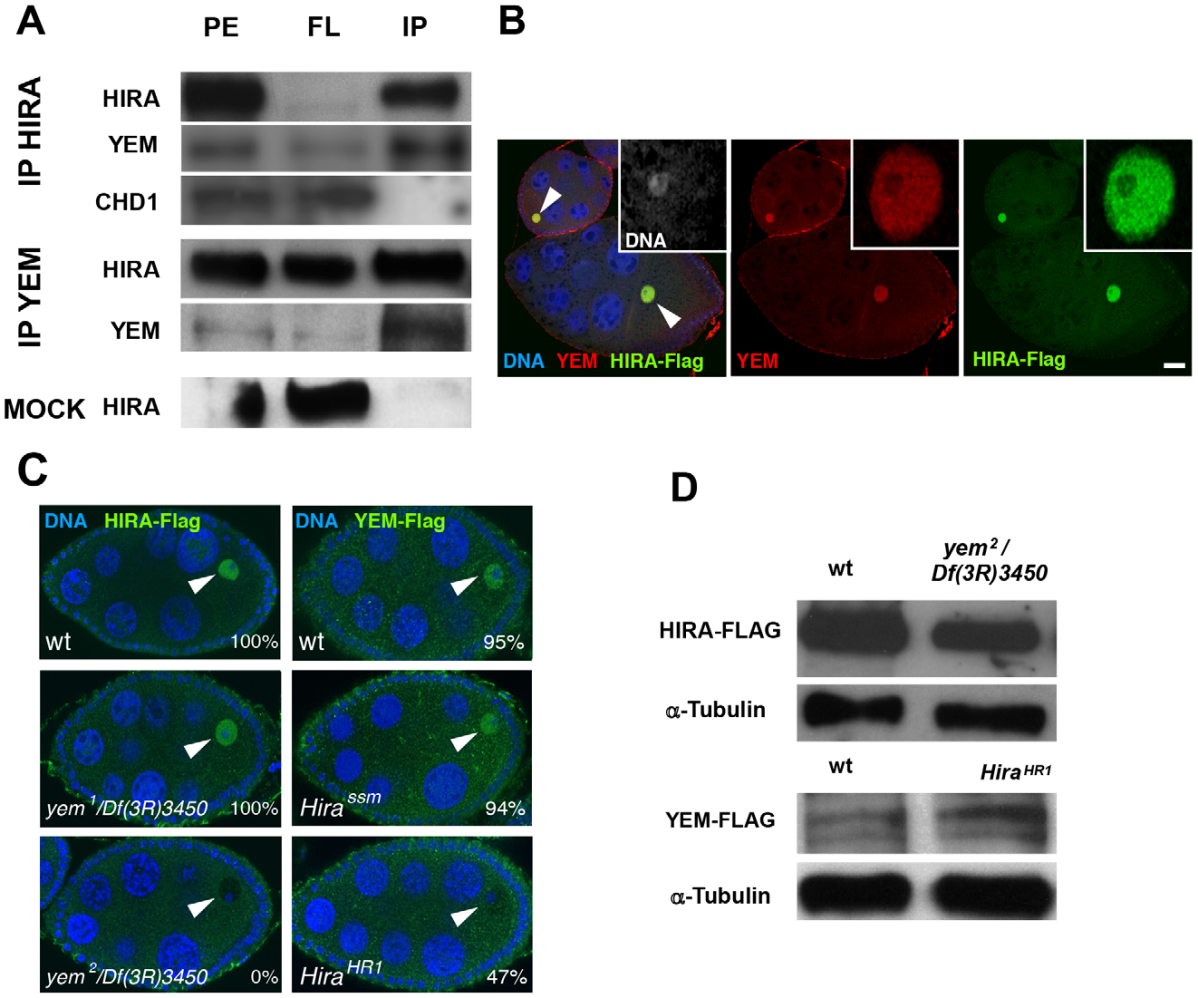
YEM and HIRA are interdependent for their localization in the germinal vesicle. (A) HIRA and YEM interact *in vivo*. Ovary extracts were prepared from *Hira-flag* transgenic flies and immunoprecipitated with either YEM AS2 polyclonal antibody or anti-Flag monoclonal antibody in the conditions described in the Materials and Methods section. The mock immunoprecipitation was performed with a rabbit pre-immune serum. The protein extracts (PE), the flowthrough (FL) and the immunoprecipitated (IP) fractions were submitted to Western blot analysis. HIRA and YEM were revealed respectively with anti-Flag (1∶1000) and AS2 (1∶100) antibodies. Rabbit polyclonal antibody against CHD1 was used at a 1∶250 dilution. Note that YEM and HIRA were found in the same immune complex whereas CHD1 was recovered in the FL fraction. In the mock experiment, HIRA was recovered in the FL fraction, which assesses the specificity of the immunoprecipitation reactions. (B) YEM and HIRA colocalize in the GV (arrowheads) throughout oogenesis. Wild-type ovaries dissected from *Hira-Flag* transgenic females were stained with anti-Flag (green), anti-YEM antibodies (red) and DAPI (blue). Bar: 20 µm. (C) YEM and HIRA proteins are interdependent for their localization in the GV. Ovaries from wild-type (wt), *yem^1^/Df(3R)3450* or *yem^2^/Df(3R)3450* females bearing one copy of the *Hira-Flag* transgene were stained to visualize DNA (blue) and Flag (green). HIRA accumulation in the GV (arrowheads) is not affected in *yem^1^/Df(3R)3450* but is abolished in *yem^2^/Df(3R)3450* ovaries. Conversely, YEM-Flag GV localization was not significantly affected in *Hira^ssm^* ovaries, but appeared highly variable in *Hira^HR1^* ovaries. Percentages indicate the number of egg chambers with positive staining in the GV, equal or superior to the background fluorescence. At least 60 egg chambers were observed for each experiment. (D) Western-blot analysis of HIRA-Flag (left) and YEM-Flag (right) from protein extracts of ovaries of the indicated genotypes. α-Tubulin was used as a loading control.

The YEM protein has been previously detected in a HIRA complex purified from embryonic nuclear extracts [53], suggesting that it could represent the *Drosophila* ortholog of UBN1/Hpc2. To more directly test the interaction of HIRA and YEM, we performed co-immunoprecipitation experiments using functional Flag-tagged and GFP-Flag-tagged transgenic versions of YEM and HIRA proteins, respectively. We confirmed that, in ovarian protein extracts, HIRA was able to co-immunoprecipitate with YEM, and vice versa (Figure 2A). In the same experiments, however, the ATP-dependent chromatin remodeling factor CHD1 was not detected in the HIRA immune complex, in contrast to what was previously reported [54]. Although the reason for this apparent discrepancy with the study by Konev *et al.* is not clear, it reinforces the fact that, in our experimental conditions, HIRA and YEM show reproducible and specific interaction, confirming that these proteins are subunits of a common complex.

HIRA and YEM were previously shown to display a remarkable and specific accumulation in the nucleoplasm of the GV throughout oogenesis [36], [51]. Similarly, immunodetection of the Flag-tagged versions of HIRA and YEM recapitulates their endogenous accumulation in the GV, where both proteins co-localize (Figure 2B). The oocyte nucleus is a large nucleus that essentially contains nucleoplasm, as the oocyte chromosomes remain confined within a small, compact structure called the karyosome [55]. Surprisingly, we observed that HIRA-Flag accumulation in the GV was completely abolished in *yem^2^*/*Df(3R)3450* mutant oocytes. Conversely, we found that YEM-Flag was undetectable in the GV of about half of null *Hira^HR1^* mutant oocytes (Figure 2C). These effects could not be explained by reduced protein levels in mutant flies, as HIRA-Flag and YEM-Flag expression were apparently not affected in *yem^2^/Df(3R)3450* an *Hira^HR1^* mutants, respectively (Figure 2D). These results indicate that YEM and HIRA are mutually required for their localization or for their stabilization in the oocyte and suggest that these proteins interact prior to their release in the egg cytoplasm, after GV breakdown. Taken together, these results confirm that YEM and HIRA belong to the same complex *in vivo*.

### YEM is required for paternal chromatin assembly at fertilization

**Figure 3:**
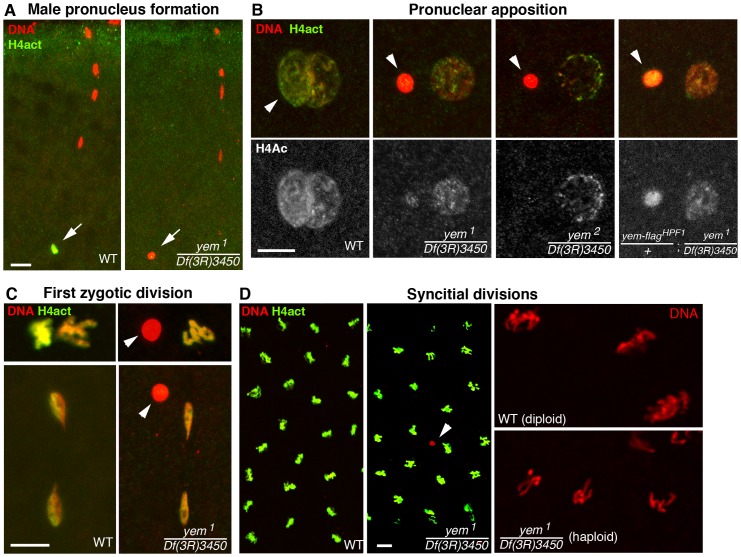
YEM is essential for chromatin assembly in the male pronucleus at fertilization. (A) Confocal images of fertilized eggs in telophase of meiosis II stained for DNA (red) and acetylated histone H4 (H4act, green). In wild-type eggs (WT), the male pronucleus (arrow) is brightly and specifically stained with anti-H4act antibodies. In *yem* mutant eggs, H4act incorporation in the male nucleus is very weak or absent. (B) Eggs at the pronuclear apposition stage. In *yem^1^* and *yem^2^* mutant eggs, the male pronucleus (arrowheads) fails to decondense (compare with wild-type) and contains very low or undetectable levels of H4act. Note that paternal chromatin assembly is partially restored in eggs laid by weakly fertile *yem-flag^HPF1^/+; yem^1^/Df(3R)3450* females (see also Table 1). (C) Cycle 1 embryos in metaphase (top) and telophase (bottom), stained as in (A) and (B). In *yem* mutant eggs, the male nucleus (arrowheads) is excluded from the first zygotic division. (D) *yem^1^/Df(3R)3450* females produce gynogenetic haploid embryos. Left: the male nucleus (red) is still detected (arrowhead) among the haploid cleavage nuclei containing chromosomes of maternal origin. Right: close-up of cleavage nuclei in metaphase from wild-type (diploid) and *yem* (haploid) embryos.

The female sterility associated with *yem^1^* or *yem^2^* mutations actually results from a maternal effect embryonic lethality phenotype. Indeed, eggs from *yem^1^*/*Df(3R)3450* or *yem^2^*/*Df(3R)3450* females (referred to as *yem* mutant eggs for simplicity) are normally fertilized and they initiate development, but the embryos systematically die before hatching (Table 1 and not shown). These features are reminiscent of the maternal effect embryonic lethality phenotype of *Hira* mutants, where embryos develop as non-viable gynogenetic haploids after the loss of paternal chromosomes during the first zygotic division [36], [37], [56]. We thus examined male pronucleus formation in *yem* mutant eggs. In wild-type eggs, shortly after fertilization, while maternal chromosomes complete meiotic divisions, the decondensing male nucleus is strongly and specifically stained with an antibody recognizing acetylated histone H4, a mark of newly assembled chromatin [36], [37]. Strikingly, we observed that in *yem* mutant eggs, acetylated H4 was practically not incorporated in the male pronucleus (Figure 3A). At pronuclear apposition, male pronuclei in *yem* mutant eggs always appeared round and condensed (Figure 3B), in a way identical to the male nucleus in *Hira* mutants [36], [37], [57]. Paternal chromosomes subsequently failed to integrate the first zygotic division in *yem* eggs (Figure 3C), resulting in gynogenetic haploid development and embryonic lethality (Figure 3D). It should be mentioned however that exceptional gynogenetic development of adults can occur if the female pronucleus is diploid as the result of defective meiosis [52].

While the *yem-flag^HPF16^* transgene efficiently rescued *yem* female sterility, another insertion of the same construct (*yem-flag^HPF1^*) only restored fertility to very low levels, likely because of its weak expression (Table 1). Interestingly, in eggs laid by *yem^1^/Df(3R)3450; yem-flag^HPF1^* females, the male pronucleus still appeared round and condensed but consistently incorporated significant levels of acetylated histone H4 (Figure 3B). This suggests that the level of maternal YEM protein is limiting for both nucleosome assembly and male pronucleus decondensation.

**Figure 4:**
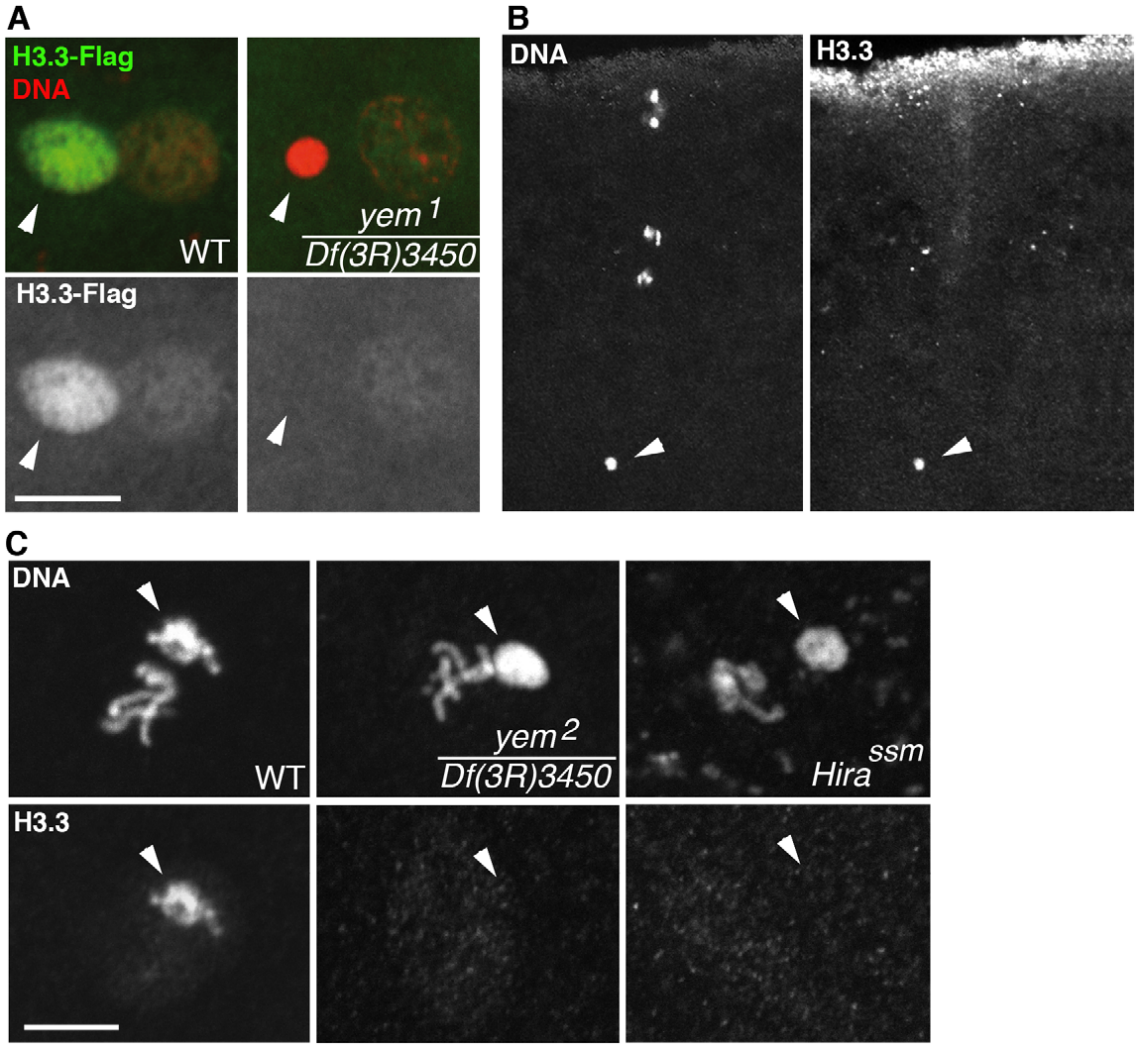
*yem* is required for H3.3 deposition in the male nucleus. (A) At pronuclear apposition in wild-type eggs, the male nucleus (arrowhead) contains high levels of maternally expressed H3.3-Flag whereas the female pronucleus incorporates low levels of H3.3-Flag, presumably during S phase. In *yem^1^/Df(3R)3450* mutant eggs, only the weak incorporation of H3.3-Flag in the female pronucleus is detected. (B) The monoclonal anti-H3.3 antibody specifically stains the male nucleus (arrowhead) at fertilization in wild-type eggs. Maternal chromosomes are in anaphase of the second meiotic division. (C) H3.3 is still detected in paternal chromosomes (arrowheads) during the first zygotic cycle in wild-type eggs but not in the male nucleus on cycle 1 *yem* or *Hira* mutant embryos. Bars: 10 µm.

We have previously shown that HIRA-dependent nucleosome assembly in the male pronucleus exclusively uses the histone H3 variant H3.3 [36], [37]. To observe H3.3 deposition in the male pronucleus, we used a previously described, maternally expressed Flag-tagged transgenic version of H3.3 (H3.3-Flag) [37]. In contrast to control eggs, H3.3-Flag was not incorporated in paternal chromatin of *yem^1^* eggs, similarly to *Hira* mutants (Figure 4A). However, the female pronucleus in *yem* eggs still incorporated low levels of H3.3-Flag during the first round of DNA replication, arguing that, like HIRA, YEM does not participate to the limited S phase deposition of H3.3 which occurs in replicating nuclei of early embryos [36] (Figure 4A). As a complementary approach, we analyzed the *yem* mutant phenotype using a commercially available monoclonal anti-H3.3 antibody. In wild-type fertilized eggs, the antibody specifically stained the decondensing male pronucleus, but not the maternal chromosomes, thus confirming its specificity for H3.3 (Figure 4B). In agreement with the results obtained with H3.3-Flag, no staining was detected above background when *Hira* and *yem* mutant eggs were stained with the anti-H3.3 antibody (Figure 4C). Altogether, these results demonstrate the critical requirement of YEM for the assembly of H3.3-containing nucleosomes on paternal DNA.

Although mutant *yem^1^*/*Df(3R)3450* and *yem^2^*/*Df(3R)3450* adults were viable, survival rates were reduced for *yem^2^*/*Df(3R)3450* individuals (Table S1) indicating that YEM also functions in somatic cells. Interestingly, the partial lethality of *yem^2^* mutant individuals was not aggravated when combined with the *Hira^HR1^* null allele. Thus, HIRA and YEM do not have redundant functions but, instead, are obligate partners not only for male pronucleus chromatin assembly but presumably also for other somatic RI nucleosome assembly processes.

### HIRA and YEM cooperate for their localization in the male pronucleus

**Figure 5:**
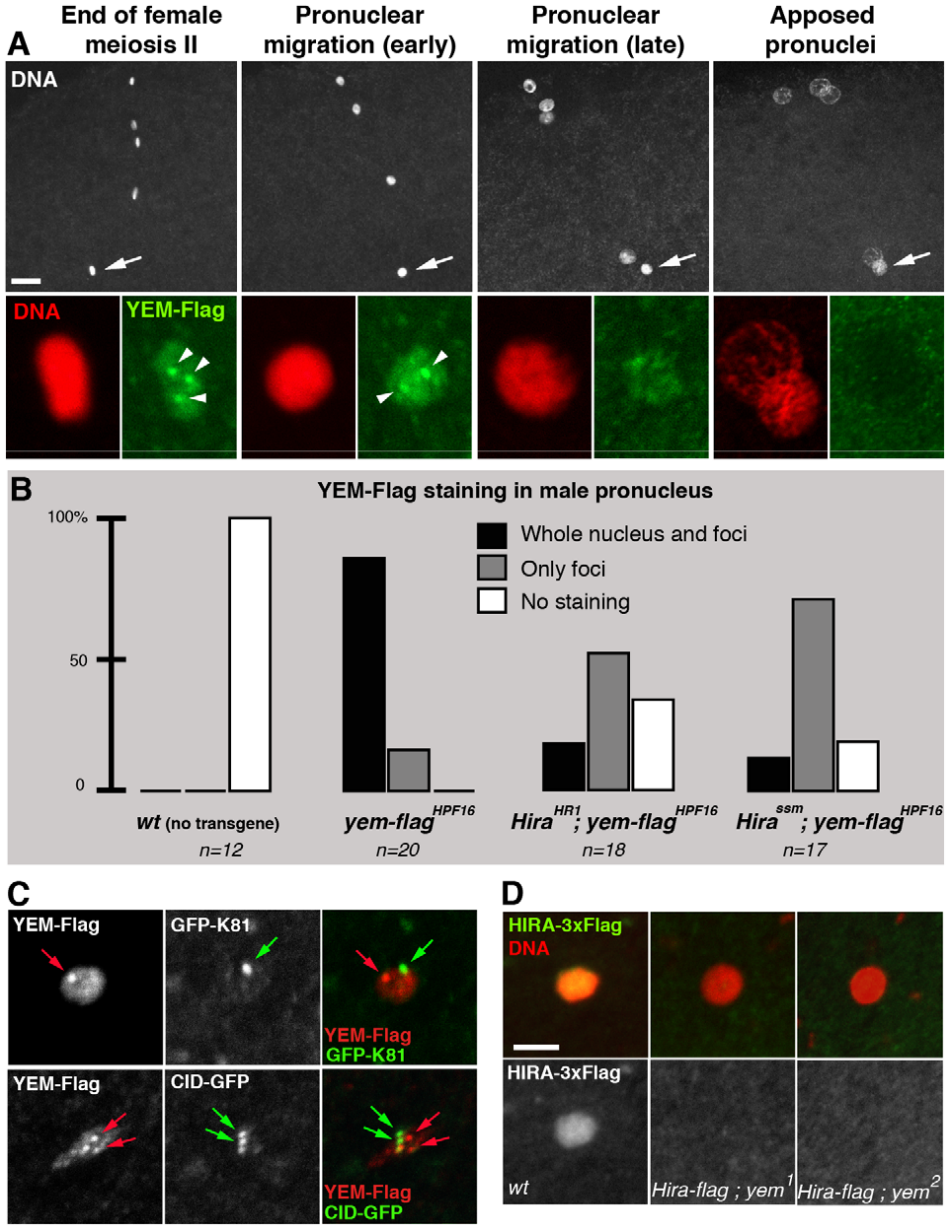
HIRA and YEM are interdependent for their recruitment to the male pronucleus. (A) YEM localizes to the decondensing male nucleus. Confocal images of eggs from *yem-flag^HPF3^ yem^2^* homozygous females (upper panels) with the male nucleus (arrows) magnified in lower panels. YEM-Flag (green) is detected throughout the decondensing male nucleus but also accumulates in a small number of nuclear foci (arrowheads). Note that YEM-Flag is no longer detected at pronuclear apposition (right panels). Bar: 10 µm. (B) *Hira* mutations affect the general distribution of YEM-Flag in the male nucleus. Eggs from females of the indicated genotype were stained with anti-Flag antibodies and imaged as in (A). For each male nucleus, the presence of YEM-Flag (whole nucleus and/or foci) was evaluated and each category is represented as a percentage of the total number (n) of observed pronuclei. (C) YEM-Flag foci in the male nucleus do not colocalize with telomeres or centromeres. Upper panels: Nuclear foci of maternally expressed YEM-Flag (red arrow) do not localize to the cluster of telomeres marked with the paternal telomere marker GFP-K81 [69] (green arrow). Lower panels: YEM-Flag nuclear foci (red arrows) do not colocalize with paternal centromeres (green arrows) in the male nucleus. (D) Confocal sections of male pronuclei in eggs laid by females of the indicated genotype (HIRA-Flag is shown in green, DNA in red) (n>20). In wild-type eggs, HIRA-Flag accumulates in the decondensing male nucleus. In eggs from *yem* mutant females however, HIRA-Flag is not detected.

Consistent with its critical role in paternal chromatin assembly, maternally expressed HIRA is recruited to the male nucleus shortly after fertilization in both *Drosophila* and mouse [37], [38]. Strikingly, while robust HIRA-Flag staining is observed in the decondensing male nucleus in control eggs, HIRA-Flag was not detected in eggs from *yem^1^* and *yem^2^* females (n>20; Figure 5D). Thus, YEM is required for the recruitment or for the stabilization of HIRA in the male nucleus. As expected, maternal YEM-Flag was also detected in the decondensing male nucleus before pronuclear apposition (Figure 5B). However, in contrast to the homogeneous distribution of HIRA-Flag in the male nucleus, YEM-Flag appeared also enriched in a small number of discrete foci of unknown nature (Figure 5A). We verified that these foci localized neither to the centromeres nor to the telomeres of the male pronucleus (Figure 5C). Interestingly, the formation of these YEM-Flag foci appeared largely independent of HIRA, whereas the rest of YEM-Flag was not detected in a large majority of *Hira* mutant eggs (Figure 5B). Thus, with the exception of these discrete regions, our experiments demonstrate that HIRA and YEM are interdependent for their localization within the male pronucleus and for paternal chromatin assembly.

### Drosophila ATRX/XNP is not involved in paternal nucleosome assembly

**Figure 6:**
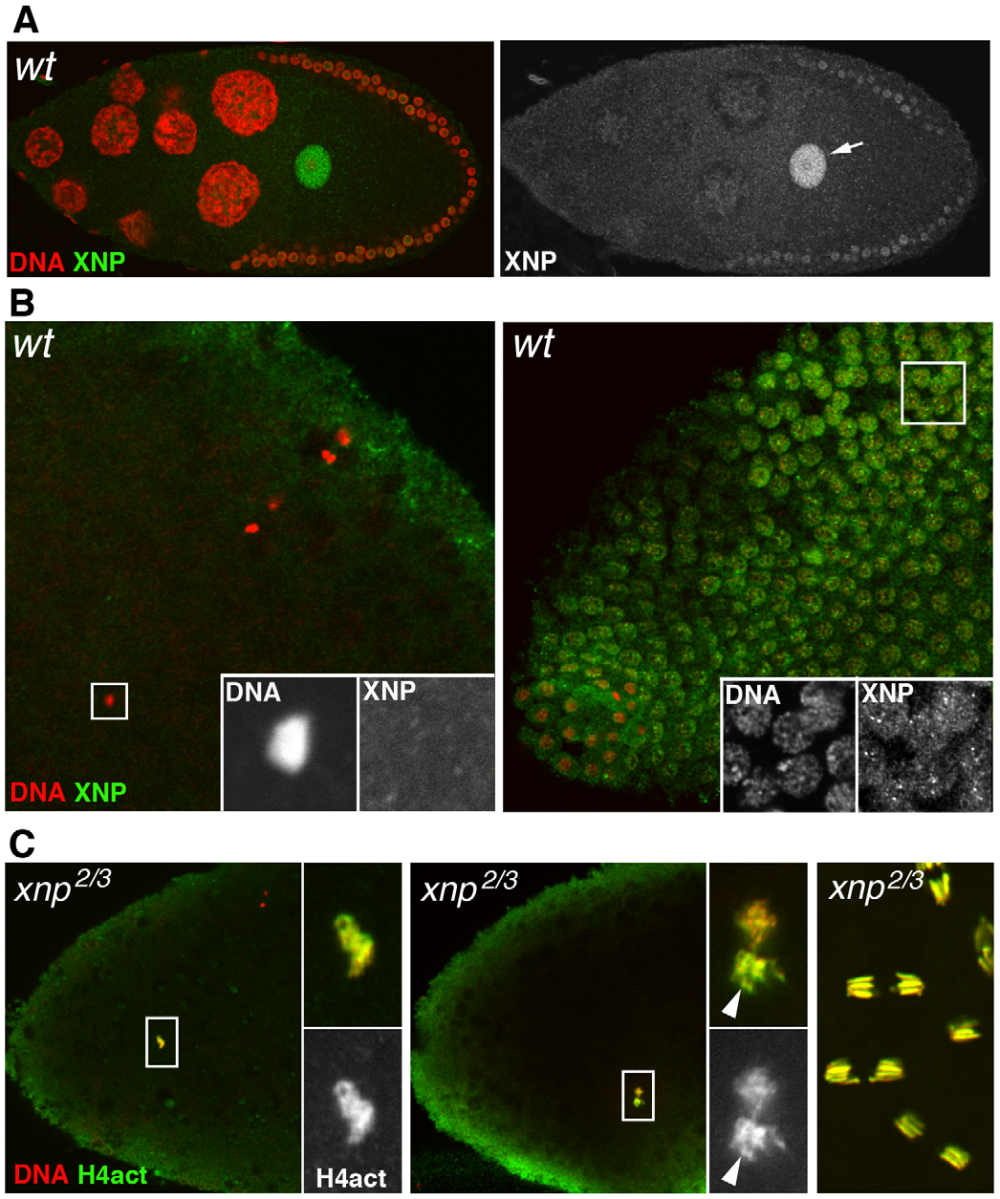
XNP is not involved in paternal chromatin assembly. (A) The dATRX/XNP protein (green) is expressed in the female germline and accumulates in the germinal vesicle (arrow) of the oocyte, here in a stage 10 egg chamber. (B) At fertilization (left panel), XNP is not observed in the male nucleus (inset) whereas it is detected in the nuclei of blastoderm embryo. (C) Confocal images of eggs or embryos from *xnp* mutant females stained for anti-H4act (green) and DNA (red). Left: in eggs from *xnp* mutant females, assembly of paternal chromatin seems to occur normally, as revealed by the strong incorporation of acetylated H4 in the male nucleus (inset). Middle: a cycle 1 embryo in prometaphase with the still separated sets of maternal and paternal chromosomes. Note the stronger anti-H4act staining of paternal chromosomes (arrowhead) relative to maternal chromosomes, as in wild-type embryos (see Figure 3C). Right: normal cleavage divisions in a syncytial embryo.

Several groups have recently established that in mammalian cells, RI H3.3 deposition is mediated by at least two distinct protein complexes. HIRA and its partners are involved in the enrichment of H3.3 at active genes and at upstream regulatory elements of both active and repressed genes [6]. In contrast, ATRX, a member of the SNF2 family of ATP-dependent chromatin remodeling factors and the histone chaperone DAXX (Death-Associated protein) are essentially responsible for the enrichment of H3.3 nucleosomes at heterochromatin loci [6], [58]–[60]. In *Drosophila*, the ATRX homolog XNP (or dATRX) colocalizes with H3.3 throughout the chromatin of somatic cells [16]. To investigate the potential involvement of this chromatin remodeler in the assembly of paternal nucleosomes in the newly fertilized egg, we first determined its distribution in oocytes and eggs using a specific antibody recognizing both XNP isoforms [16]. Interestingly, XNP was found to accumulate in the oocyte nucleus, in a way remarkably similar to HIRA and YEM (Figure 6A). However, XNP was not observed in the decondensing male nucleus at fertilization (n>20) and the protein remained absent from early cleavage nuclei until their migration to the embryo periphery, at the syncytial blastoderm stage (Figure 6B and not shown). In addition, we observed that chromatin assembly in the male nucleus occurred normally in eggs from *xnp^2^/xnp^3^* mutant females (n>20; Figure 6C). Finally, females homozygous for the semi-lethal allele *xnp^3^*, which abolishes the expression of the long XNP isoform [61], produced a limited amount of eggs that nevertheless hatched (not shown). We conclude that dATRX/XNP is most likely not involved the assembly of paternal nucleosomes at fertilization.

## Discussion

In human cells, the HIRA core complex is composed of at least three subunits, including HIRA, UBN1 and CABIN1 [43]. This complex is functionally involved in a large diversity of cellular and developmental processes that require dynamic histone turnover or *de novo* assembly of nucleosomes, independently of DNA synthesis. Although the HIRA complex mediates the deposition of the highly conserved H3.3 histone variant, its subunits display a comparatively weak overall conservation in animals. For instance, *Drosophila* does not seem to have any CABIN1 homolog and the highest conservation between UBN1 and YEM is mainly restricted to the small HRD domain. Despite this poor conservation, our work establishes Yemanuclein as a *bona fide* ortholog of Ubinuclein, by demonstrating its physical interaction with the HIRA histone chaperone and its critical requirement for H3.3 deposition during male pronucleus decondensation.

### Paternal chromatin assembly is a major function of YEM

In contrast to the knock-out of the *Hira* gene in mouse, which is zygotic lethal in early embryos [62], null mutants of Drosophila *Hira* are viable but homozygous females are completely sterile [36]. This indicates that only the maternal contribution of *Hira* is essential, at least to form the male pronucleus. Our characterization of a null *yem* ^2^ allele allowed us to reach the same conclusion for YEM. Remarkably, the phenotype of the male pronucleus in eggs laid by *yem* mutant females appeared indistinguishable to what we previously reported for *Hira* mutants. In both cases, RI deposition of H3.3-containing nucleosomes is practically abolished, typically preventing the full decondensation of the male nucleus and its integration into the zygotic nucleus. Thus, YEM and HIRA are equally required to assemble paternal nucleosomes at fertilization. This unique and major function of the HIRA complex is most likely conserved in animal groups where histones, and notably H3 and H4, are replaced with SNBPs in sperm. This is for instance the case of mammals, where protamines package about 95% and 85% of mouse and human sperm DNA, respectively [30], [32]. In fact, HIRA has been previously detected in the decondensing male nucleus at fertilization in mouse, which incorporates H3.3 before the first round of DNA replication [38], [39]. We thus expect Ubinuclein1/2 to be also involved in paternal chromatin assembly in mammals. In apparent contradiction with this prediction, a transgene expressing human UBN1 in the female germline could not rescue the sterility of *yem* mutant females (Figure S1 and not shown). However, this absence of complementation of YEM and UBN1 can be explained by the strong divergence of these orthologous proteins at the primary sequence level and it suggests that UBN1 can only function within its native, human HIRA complex. The apparent lack of a CABIN1 homolog in *Drosophila* also underlines the central role played by the HIRA-UBN1/YEM pair in the complex. Interestingly, while the implication of HIRA and UBN1 for RI deposition of H3.3 *in vivo* was recently demonstrated in human cells, CABIN1 seemed to play only an auxiliary role in this context [63]. Possibly, CABIN1 could be important for human-specific functions of the HIRA complex, such as the formation of senescence-associated heterochromatin foci [45], [64].

### HIRA and YEM are interdependent to target the male nucleus

We had previously shown that HIRA specifically accumulates in the sperm nucleus shortly after its delivery in the egg cytoplasm [37]. Here, we have established that maternally expressed YEM similarly accumulates in the male nucleus at fertilization and until pronuclear apposition. Strikingly, we have also shown that HIRA and YEM are mutually dependent for their targeting to the male nucleus, strongly suggesting that these proteins physically interact during the assembly of paternal nucleosomes. However, nothing is known about the mechanism responsible for their rapid and specific localization in the fertilizing sperm nucleus, which is delivered in the cytoplasm of the gigantic egg cell. We had previously established that the HIRA-dependent assembly of paternal nucleosomes occurs after the removal of sperm protamines [36]. This opens the simple possibility that the HIRA complex could recognize exposed sperm DNA immediately after the removal of SNBPs. Interestingly, pioneer work on YEM by Aït-Ahmed et al. had established that this maternal protein was able to bind DNA *in vitro* [51]. This property could be important to efficiently target the HIRA complex to sites of *de novo* nucleosome assembly in the decondensing male nucleus. This hypothesis has recently received indirect experimental support in human cultured cells [63]. In their study, Ray-Gallet *et al.* established that HIRA, UBN1 and CABIN1 were all individually able to bind DNA *in vitro* and they proposed that this remarkable property could allow the HIRA complex to target naked DNA for H3.3 deposition. Accordingly, this HIRA-dependent nucleosome gap-filling mechanism has been shown to participate in the maintenance of genome integrity [63], but could also be employed, at the genome-wide scale, for *de novo* assembly of paternal chromatin at fertilization.

Finally, the observation that YEM accumulates in discrete nuclear regions in both the male nucleus (this study) and the oocyte karyosome [52] opens the possibility that YEM could perform additional roles not related to nucleosome assembly.

### Paternal chromatin assembly is specifically performed by the HIRA-YEM complex

Despite its expression in the female germline, we found that *Drosophila* ATRX/XNP is not targeted to the male nucleus and does not seem to play any role in male pronucleus formation. Among the 17 SNF2 type chromatin remodelers present in *Drosophila* [16], the Chromodomain-helicase-DNA-binding protein 1 (CHD1) is the only one that has been implicated in the remodeling of paternal chromatin at fertilization [21], [54]. In contrast to *Hira* and *yem*, mutations in *chd1* do not drastically affect H3.3 incorporation in paternal chromatin but still severely compromise the decondensation of the male nucleus, which appears aberrant in shape [21], [54]. In contrast to the HIRA/CHD1 interaction reported by Konev *et al.* [54], we could not detect any interaction between these proteins in ovaries, using experimental conditions that permitted co-immunoprecipitation of HIRA and YEM. Our results thus suggest that the role of CHD1 in the male nucleus is distinct from the nucleosome assembly process mediated by the HIRA complex.

Although the implication of the HIRA histone chaperone in paternal chromatin assembly was firmly established a few years ago, it has remained unclear until now if this highly specialized RI assembly process also involved other subunits of the HIRA complex or other histone deposition pathways. In fact, we have previously reported that the histone chaperone ASF1 [65], which is known to interact with both the CAF1 and HIRA complexes, was actually absent from the decondensing male nucleus [36]. Although the role, if any, of ASF1 in paternal chromatin assembly awaits a proper functional characterization, we do not expect this histone chaperone to be directly involved in the assembly of nucleosomes on paternal DNA. Accordingly, ASF1 has been previously shown to be dispensable for direct *de novo* RC or RI histone deposition in *Xenopus* egg extracts [66].

The complete failure of the male nucleus to assemble its chromatin in *Hira* or *yem* mutant eggs demonstrates that no other nucleosome assembly machinery can substitute for the HIRA-YEM complex in this peculiar context. However, the functional requirement of H3.3 itself in this process is not known. In *Drosophila*, H3.3 is not absolutely required for survival but it is essential for both male and female fertility [27], [28]. Viability of *His3.3A; His3.3B* double null mutants could be explained by the fact that, in the absence of H3.3, canonical H3 can be assembled in a RI manner [28]. Although the mode of RI deposition of replicative H3 in these mutants is not known, it opens the possibility that HIRA could use canonical H3 in certain critical circumstances, such as a limiting availability of H3.3. This compensatory mechanism, however, is apparently not possible in *Drosophila* spermatocytes, where H3.3 is required for the correct segregation of chromosomes during meiotic divisions, underlining the importance of this variant for sexual reproduction [28]. Similarly, future work should aim at determining whether H3.3 is specifically required for the assembly of paternal nucleosomes at fertilization.

### The Germinal Vesicle: A storage compartment for maternal nuclear proteins?

Both HIRA and YEM proteins, which are presumably expressed from germinal nurse cells, display a remarkable accumulation in the oocyte nucleus during oogenesis [36], [51]. Most of the volume of the large germinal vesicle is devoid of DNA as the maternal genome is tightly packaged within the karyosome. The presence of HIRA and YEM in the nucleoplasm of the GV is thus not related to nucleosome assembly. However, the fact that HIRA and YEM are mutually dependent for their accumulation in the GV suggests that they are stored in this compartment as a complex. In contrast to the null alleles, point mutations do not affect HIRA/YEM localization in the GV, suggesting that the mechanisms controlling their recruitment to the GV or to the male pronucleus are distinct. This could reflect the fact that the HIRA complex is active in the male pronucleus where these proteins are in a chromatin environment in contrast to their nucleoplasm distribution in the GV. Whether or not this transient accumulation of HIRA/YEM in the GV plays any role in the maturation of the complex before paternal chromatin assembly at fertilization remains to be tested. Interestingly, it has been proposed that in human cells, formation of senescence-associated heterochromatin foci by HIRA requires its prior localization to promyelocytic leukemia nuclear bodies, suggesting that these structures could participate in the formation of the HIRA complex before its translocation to chromatin [48], [67]. It should be mentioned, however, that dATRX/XNP also accumulates in the GV despite its dispensability for paternal chromatin assembly. A recent study [68] reported the presence of several nuclear proteins in the GV with no known function in the oocyte, suggesting that this structure could serve as a storage compartment for a large number of nuclear proteins.

In conclusion, our characterization of *Drosophila* Yemanuclein demonstrates that this protein is a functional partner of HIRA *in vivo*. It also establishes that HIRA and YEM directly cooperate in the male nucleus for the genome-wide replacement of sperm protamines with H3.3-containing nucleosomes. The specific requirement of the HIRA complex in this unique developmental chromatin assembly process implies the existence of specific properties not shared with other H3.3-deposition pathways. In this regard, future work should explore the potentially conserved DNA binding property of the HIRA complex [51], [63] and its potential role in targeting the fertilizing sperm nucleus in animals.

## Materials and Methods

### Flies

Flies were grown in standard conditions at 25°C. The *w^1118^* stock was used as a wild-type control in all experiments. The *Hira^ssm^* and *Hira^HR1^* alleles and the *Hira-flag* transgenic constructs have been described earlier [36], [37]. For the construction of the *Hira-GFP-FLAG* fusion gene, the eGFP coding sequence was inserted between the *Hira* and Flag tag sequences of *PW8-Hira-3xflag* [37]. The *yem^1^* mutation is a T>A substitution falling in the fifth exon of *yem* which results in a V478E mutation [51], [52]. The GFP-K81 transgene is described in [69]. To mark paternal telomeres we used *w^1118^/Y; 5′K81-GFP::K81; K81^2^* males [69]. The *w; P[w+, g-EGFP-cid]III.2* [70] stock has been kindly provided by Stefan Heidmann. The *Df(3R)3450* deficiency, the *P{EPgy2}EY23024* insertion and the *xnp^2^ and xnp^3^* mutant alleles [61] were obtained from the Bloomington Drosophila Stock Center.

### Generation of the *yem* deletion allele

The *yem^2^* mutation was isolated after standard remobilization of the *P{EPgy2}EY23024* element and selected for its non-complementation of the *yem^1^* chromosome. *yem^2^* is a 3180 bp deletion from position +2 in the 5′UTR (positions 24945416 to 24948596 in the genome), uncovering the first 5 exons and part of exon 6 of the *yem* gene. Note that we only refer in this study to the original gene model [51] identified as RA in Flybase (Flybase ID# FBtr0085415) and not to the recently predicted longer RB transcript (see Flybase.org).

### RT–PCR

Total RNAs were extracted with the Trizol method (Invitrogen) from at least 50 whole adults, ovaries or carcasses. Reverse transcription was performed using oligo(dT) primers and the SuperScript First-Strand Synthesis system for RT-PCR (Invitrogen). For the *yem* and *RP49* PCR reactions, the following primers were used YEMAPRIMER15/YEMAPRIMER16 and RP49FWD/RP49REV (see primers section).

### Transgenic constructs

#### *yem-flag*

The *yem* EcoRI genomic fragment in bluescript vector [51] was digested with *NheI* and *XbaI*. This fragment was replaced by a PCR amplification product with primers OA37 and OA38, bearing the Flag tag sequence in 3′ of *yem*. Next, the resulting vector was digested with *EcoRI* and *XbaI* and the *yem-flag* fragment was inserted into the Casper vector. Finally, SV40 polyadenylation signals were added to the previous construct as a *XbaI-PstI* fragment from the pCasper{AUG-βgal} plasmid [71]. The resulting transgenesis construct is called HPF (for HoloProtein flanked with Flag). HPF1 (chromosome X), HPF16 (chromosome 2) and HPF3 (chromosome 3) are independent insertions of HPF. The *yem-flag^HPF3^ yem^2^* chromosome was obtained by meiotic recombination. *yem-flag^HPF3^ yem^2^* homozygous females (used in Figure 5) are fully fertile (not shown).

#### *pUASP-Ubn1-86F*

A human *Ubn1* cDNA [47] was cloned into a *BamHI* site of the pUASP-attB vector [69]. Transgenic lines were established using the *phiC-31* integrase method [72], [73] in the M{3xP3-RFP.attP}ZH-86Fb attP platform in polytene region 86F.

### Primers

YEMAPRIMER2: TGCGAAAACCGCGACCAGTG

YEMAPRIMER9: GGGCAGTTGTTGCGTGGATG

YEMAPRIMER15: GGATCCCATTCCTCCGCTTG

YEMAPRIMER16: CTCAGGCAGCAGCACTCAAT

RP49FWD: AAGATCGTGAAGAAGCGCAC

RP49REV: ACTCGTTCTCTTGAGAACGC

OA37: ACGTCCAAGCAGCTAGCTGCCA

OA38: GAATCTAGACTTGTCATCGTCGTCCTTGTAGTCTTGGCGCGTGGGCGTACT

### Immunofluorescence

Eggs were collected, dechorionated, devitellinized and fixed in methanol as described [56]. Eggs were then rehydrated in TBS-Triton 0,15% and incubated with primary and secondary antibodies at the indicated dilution. Finally, eggs were incubated in a 2 mg/ml RNAse A solution for 1 h at 37°C and were mounted in a mounting medium (DAKO S3023) containing 5 µg/ml propidium iodide. For anti-YEM AS2 antibody staining, ovaries were dissected in PBS-Triton 0,1% and were immediately incubated with the antiserum without fixation, stained with DAPI and mounted, as described [74]. For other experiments, ovaries were dissected in PBS-Triton 0,1% and fixed at room temperature in 4% PFA in PBS for 25 minutes. Ovaries were then stained with propidium iodide and mounted as described above. Slides were observed under an LSM 510 META confocal microscope (Zeiss). Images were treated with LSM image browser, Image J or Photoshop CS2 (Adobe).

We used the following antibodies: AS2 anti-YEM antibody (1/100; [51], [74]), M2 monoclonal anti-Flag antibody (1∶500 in ovaries, 1∶1000 in embryos; Sigma), anti-polyacetylated histone H4 (1∶200; Millipore 06-589), monoclonal anti-H3.3 (H3F3B) (1∶800, Abnova), anti-XNP [16] (1∶5000) and anti-UBN1 (1∶200) [75]. Secondary antibodies were Alexa488 goat anti-mouse or goat anti-rabbit (1∶1000, Invitrogen) and Cy3 donkey anti-rabbit (1∶800, Millipore).

### Western blots

50 µl of ovaries were homogenized in lysis buffer (15 mM Hepes (pH 7.6); 10 mM KCl; 5 mM MgCl2; 0.5 mM EDTA; 0.5 mM EGTA; 350 mM Sucrose; 1 mM DTT) with protease inhibitors (Halt Protease Inhibitor Single Use Cocktail, Thermo Scientific; 1 mM PMSF). The protein extract was centrifuged, isolated from debris and stocked in half volume of glycerol at −80°C. SDS-Page electrophoresis was carried out on 8% acrylamide gels and western blot was performed using standard procedures using Pierce ECL Western Blotting Substrate (Thermo Scientific). The following antibodies were used: M2 anti-Flag (1∶1000; Sigma), anti-Tubulin (1/1000; Sigma), Peroxydase-coupled goat anti-mouse (1∶10000; Beckman).

### Immunoprecipitations

For co-immunoprecipitation experiments, we essentially used the protocol described in Jäger et al., 2001 [76] with some modifications as indicated. A hundred ovaries were dissected manually in 250 µl lysis buffer on ice. Lysis buffer was as described [76] to the exception of the protease inhibitors. In our conditions, Roche tablets of EDTA-free protease inhibitor cocktail were used as recommended by the supplier. PMSF was also added to a 1 mM final concentration. Before homogenization 250 µl ice-cold lysis buffer were added. The homogenates were cleared by centrifugation and the supernatant was adjusted to 1 ml in lysis buffer. The protein extracts were then submitted to the immunoprecipitation procedure after 2×30 µl were set aside to be used as input in western blot experiments. G-Sepharose beads (Sigma) were used as recommended by the supplier with the following antibodies at a 1/250 dilution: mouse monoclonal Flag M2 (Sigma) for HIRA and the AS2 rabbit polyclonal for YEM. Rabbit preimmune serum was used as negative control. Gel separation and western blots analysis were performed as indicated above. The rabbit CHD1 antibody (a gift from A. Lusser) was used at a 1/250 dilution. Secondary antibodies were goat peroxydase-coupled anti-mouse and anti-rabbit antibodies (1∶10000; Beckman). Revelation was performed with the Millipore Immobilon Western Chemiluminescent substrate as recommended by the supplier.

## Supporting Information

Figure S1 Immunodetection of human UBN1 in egg chambers of *Act5C-Gal4/+; pUASP-Ubn1-86F/+* females. UBN1 accumulates in the germinal vesicle (arrow).
(JPG)

Table S1 All the indicated crosses were performed under standard conditions, at 25°C in several non-crowded vials. All the progenies from each cross were considered, [Hu^+^] progenies (not carrying a balancer chromosome) were counted separately and their rate to total population was calculated (in every cross, [Hu^+^] progeny is expected to be 33% of total). For most of the crosses, this percentage exceeds 33%, showing normal viability of the *yem^EY23024^* insertion allele and of the *yem^1^* allele. The *yem^2^* allele is viable but shows lower survival rate than *yem^1^*. This sub-viability can be rescued with two *yem-flag* insertions, showing that it is indeed a specific effect of the *yem* mutation.
(DOCX)
