# Supplementary figures and images for: Drosophila Yemanuclein and HIRA Cooperate for De Novo Assembly of H3.3-Containing Nucleosomes in the Male Pronucleus
Source: PLoS Genet. 2013 Feb 7;9(2):e1003285. doi: 10.1371/journal.pgen.1003285 (PMC3567178; doi:10.1371/journal.pgen.1003285)

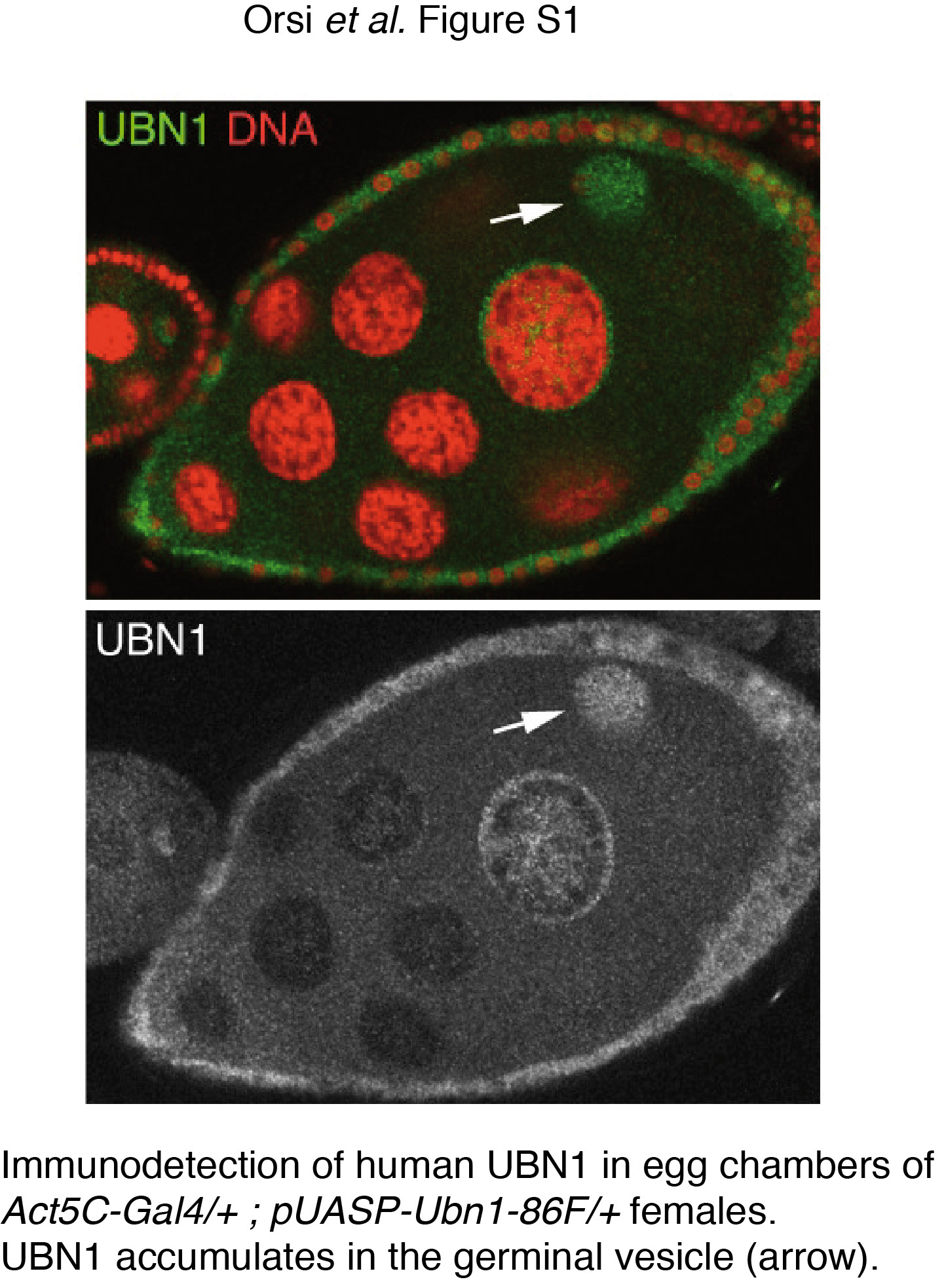

Supplement: Figure S1 — Immunodetection of human UBN1 in egg chambers of Act5C-Gal4/+; pUASP-Ubn1-86F/+ females. UBN1 accumulates in the germinal vesicle (arrow). (JPG) [file pgen.1003285.s001.jpg]
