# Supplementary material for: Drosophila Yemanuclein and HIRA Cooperate for De Novo Assembly of H3.3-Containing Nucleosomes in the Male Pronucleus
Source: PLoS Genet. 2013 Feb 7;9(2):e1003285. doi: 10.1371/journal.pgen.1003285 (PMC3567178; doi:10.1371/journal.pgen.1003285)
Supplement: Table S1 — All the indicated crosses were performed under standard conditions, at 25°C in several non-crowded vials. All the progenies from each cross were considered, [Hu+] progenies (not carrying a balancer chromosome) were counted separately and their rate to total population was calculated (in every cross, [Hu+] progeny is expected to be 33% of total). For most of the crosses, this percentage exceeds 33%, showing normal viability of the yemEY23024 insertion allele and of the yem1 allele. The yem2 allele is viable but shows lower survival rate than yem1. This sub-viability can be rescued with two yem-flag insertions, showing that it is indeed a specific effect of the yem mutation. (DOCX) [file pgen.1003285.s002.docx]

| **Table S1. Viability of the *yem* mutant alleles** | | |
| --- | --- | --- |
| **Crosses** | **No. of progeny** | **[Hu+] progeny (%)** |
| *♀♀ w ; yem^EY23024^/TM6* X *♂♂ w/Y ; yem^EY23024^/TM6* | 265 | 101 (38.1) |
| *♀♀ w ; yem^2^/TM6* X *♂♂ w/Y ; yem^2^/TM6* | 144 | 8 (5.6) |
| *♀♀ w ; yem^2^/TM6* X *♂♂ w/Y ; yem^1^/TM6* | 369 | 152 (41.2) |
| *♀♀ w ; yem^1^/TM6* X *♂♂ w/Y ; yem^2^/TM6* | 313 | 133 (42.5) |
| *♀♀ w ; Df(3R)3450/TM6* X *♂♂ w/Y ; e yem^1^/TM6* | 985 | 412 (41.8) |
| *♀♀ w ; Df(3R)3450/TM6* X *♂♂ w/Y ; yem^EY23024^/TM6* | 724 | 341 (47.1) |
| *♀♀ w ; Df(3R)3450/TM6* X *♂♂ w/Y ; yem^2^/TM6 (#1)^¥^* | 627 | 120 (19.1) |
| *♀♀ w ; Df(3R)3450/TM6* X *♂♂ w/Y ; yem^2^/TM6 (#2) ^¥^* | 137 | 28 (19.7) |
| *♀♀ w yem-flag^HPF1^/w ; Df(3R)3450/TM6* X *♂♂ w/Y ; yem^2^/TM6* | 201* | 75* (37.3) |
| *♀♀ w ; yem-flag^HPF16^/+ ; Df(3R)3450/TM6* X *♂♂ w/Y ; yem^2^/TM6* | 61* | 26* (42.6) |

* Only w^+^ progenies (that received the rescue transgene) were considered in these crosses.

^¥^ These are two independent repeats of the same experiment.

All the indicated crosses were performed under standard conditions, at 25°C in several non-crowded vials. All the progenies from each cross were considered, [Hu^+^] progenies (not carrying a balancer chromosome) were counted separately and their rate to total population was calculated (in every cross, [Hu^+^] progeny is expected to be 33% of total). For most of the crosses, this percentage exceeds 33%, showing normal viability of the *yem^EY23024^* insertion allele and of the *yem^1^* allele. The *yem^2^* allele is viable but shows lower survival rate than *yem^1^*. This sub-viability can be rescued with two independent *yem-flag* insertions, showing that it is indeed a specific effect of the *yem* mutation.
